# Mixture effects of thiamethoxam and seven pesticides with different modes of action on honey bees (*Apis mellifera*)

**DOI:** 10.1038/s41598-023-29837-w

**Published:** 2023-02-15

**Authors:** Wenhong Li, Lu Lv, Yanhua Wang, Yu-Cheng Zhu

**Affiliations:** 1 State Key Laboratory for Managing Biotic and Chemical Threats to the Quality and Safety of Agro-products/Key Laboratory of Detection for Pesticide Residues and Control of Zhejiang Province, Institute of Quality and Standard for Agro-products, Zhejiang Academy of Agricultural Sciences, Zhejiang 310021 Hangzhou, People’s Republic of China; 2 Guizhou Institute of Plant Protection, Guizhou Academy of Agricultural Sciences, Guiyang, 550006 Guizhou People’s Republic of China; 3 United States Department of Agriculture, Agricultural Research Service (USDA-ARS), 141 Experiment Station Road, Stoneville, MS 38776 USA

**Keywords:** Zoology, Environmental sciences

## Abstract

Even though honey bees in the field are routinely exposed to a complex mixture of many different agrochemicals, few studies have surveyed toxic effects of pesticide mixtures on bees. To elucidate the interactive actions of pesticides on crop pollinators, we determined the individual and joint toxicities of thiamethoxam (THI) and other seven pesticides [dimethoate (DIM), methomyl (MET), *zeta*-cypermethrin (ZCY), cyfluthrin (CYF), permethrin (PER), esfenvalerate (ESF) and tetraconazole (TET)] to honey bees (*Apis mellifera*) with feeding toxicity test. Results from the 7-days toxicity test implied that THI elicited the highest toxicity with a LC_50_ data of 0.25 (0.20–0.29) μg mL^−1^, followed by MET and DIM with LC_50_ data of 4.19 (3.58–4.88) and 5.30 (4.65–6.03) μg mL^−1^, respectively. By comparison, pyrethroids and TET possessed relatively low toxicities with their LC_50_ data from the range of 33.78 (29.12–38.39) to 1125 (922.4–1,442) μg mL^−1^. Among 98 evaluated THI-containing binary to octonary mixtures, 29.59% of combinations exhibited synergistic effects. In contrast, 18.37% of combinations exhibited antagonistic effects on *A. mellifera*. Moreover, 54.8% pesticide combinations incorporating THI and TET displayed synergistic toxicities to the insects. Our findings emphasized that the coexistence of several pesticides might induce enhanced toxicity to honey bees. Overall, our results afforded worthful toxicological information on the combined actions of neonicotinoids and current-use pesticides on honey bees, which could accelerate farther comprehend on the possible detriments of other pesticide mixtures in agro-environment.

## Introduction

Neonicotinoid insecticides have been widely used for over three decades, possessing 25% of the global insecticide market^1^. They are acetylcholine mimics and act as agonists of nicotinic acetylcholine receptor (nAChR), which successively stimulate activate the cholinergic receptors, results in hyper-excitation and death of insects^2,3^. Besides, these types of insecticides are frequently applied as soil drenches, seed coating or granules, which may result in their translocation and distribution throughout the whole plant due to their systemic characteristics, conferring a substantial and long-lasting control of insects and protecting growing plants^4^. The application of such direct treatments has distinct environmental advantages over widespread spray applications^5^. However, the residues of neonicotinoids can be potentially present in substrates attractive to insect pollinators, such as nectar, pollen or aphid honeydew^6,7^. Neonicotinoids influence the central nervous system of organisms, and fain to distinguish between the target (e.g. rice planthopper, aphid) and non-target insects (e.g. bees)^8,9^. As a classical neonicotinoid insecticide, thiamethoxam (THI) has become the most widely used insecticide around the world, and it is more closely related to honey bee health issues compared with other neonicotinoids^10^. Thus, the side-effects of it on insect pollinators should be examined to prevent unintended damage from thiamethoxam.

Honey bees (*Apis mellifera*) are the most economically crucial group of insect pollinators all over the world, pollinating both wildflowers and agricultural crops while producing bee products with a high economic value at the same time^11,12^. However, the overwintering losses of *A. mellifera* colonies have risen beyond the level of 20% during the last decades in many countries^13,14^. The declines may possess a potential threat to food production and the diversity of natural ecosystems^15,16^. There is an increasing attention upon the declines of pollinators in several regions of the world, especially concerning the increasing demands for pollination services^17^. Many stressors may contribute to honey bee losses, including pathogens, parasites, malnutrition, habitat fragmentation and pesticide exposure^18,19^. Honey bees can be deemed an insect specifically sensitive to pesticides, since there are fewer genes encoding xenobiotic detoxification enzymes in its genome relative to other insects^20^. In particular, the application of neonicotinoid insecticides, which has been substantially increased on a global scale over the last decade, is suspected to represent a significant factor responsible for losses of honey bee colonies^18^.

The toxicity of single compounds to pollination insects has been carried out by most of the previous studies^21^. However, pesticides are rarely found as individual chemicals in the environments^22^. On the contrary, they are often detected as mixtures^23^. Moreover, foraging bees are often contacted to multiple chemicals in agro-ecosystems^24^. Some studies have uncovered the existence of over 130 different pesticides in over 1300 wax, pollen and bee samples collected across the United States of American and Canada, with an average of six determinations each sample^15,25^. The joint toxicity of multiple pesticides has become an important safety concern because they can produce more significant negative effects than their individual constituents^26^. Because of the extensive use of pesticides, THI and other chemicals with different modes of action (MOA) are often observed in the same nectar and pollen samples^23,24^. Nonetheless, the joint toxicities of these pesticides remain mostly unexplored, which may have a potential threat to *A. mellifera*^27^. In order to protect the health of pollination insects, we assessed the interactive effects of THI and current-use pesticides on honey bees in this work.

## Materials and methods

### Test organisms

Queens and colonies of honey bees (*A. mellifera*) were bought from a local beekeeper and kept at the apiaries in the Stoneville Wildlife Management Area (33° 42′ N, 90° 91′ W, Mississippi, USA). Each test colony incorporated a young normal egg-laying queen and a working population of nine frames of comb with larvae, pupae, honey and pollen. Honey bee colonies were reared as previously described^28^. Each hive was equipped with a bottom board oil trap (35 × 45 cm tray filled with vegetable oil) for monitoring and control of *Varroa* mite (*Varroa destructor*).

### Test pesticides

The field-relevant formulations of pesticides were used to evaluate the toxic effects on *A. mellifera*. Formulated pesticides rather than their active ingredient were used because we purposed to simulate field situations and evaluate the potential interactive effects of chemical mixtures on honey bees from formulations commonly applied under field environments. Eight pesticides with five kinds of chemicals were assessed in this assay, which are widely used in the management of key agricultural pests and diseases globally, including one neonicotinoid insecticide THI (Centric 40 WG, Syngenta), one organophosphate insecticide dimethoate (DIM, Dimethoate 4 E 43.5%, Cheminova), one carbamate insecticide methomyl (MET, Lannate 2.4 LV, DuPont), four pyrethroid insecticides (*zeta*-cypermethrin (ZCY, Mustang Max/Respect 9.6%, FMC), cyfluthrin (CYF, Tombstone 2 EC, Loveland), permethrin (PER, Arctic 3.2EC, Winfield Solutions LLC) and esfenvalerate (ESF, Asana XL 0.66 EC, Bayer)) and one triazole fungicide tetraconazole (TET, Domark 230 ME, Valent). The selected eight pesticides were kept in a refrigerator (6 ± 1 °C).

### Toxicity bioassay of individual pesticide

Feeding toxicity test with adult bees emergenced for 4-days was conducted as previously reported^29^. Specifically, the pesticides were incorporated into 20 mL 50% sucrose solution to their final concentrations. Four to six concentrations with a geometrical ratio were determined to acquire median lethal concentration (LC_50_) of each chemical. Similarly, 50% sucrose solution incorporating no chemical was adopted as the control. Each treatment consisted of three replicates (cages) and each cage incorporated 20 honey bee workers. Individuals that were ataxic or unable to right themselves were scored as dead. Mortality was registered after exposure for 2, 4 and 7 days.

### Joint toxicity determination

The joint toxicities of THI and other seven pesticides were performed with adult worker bees (4 days old). To directly contrast the toxicities of single chemicals with their combinations, simultaneous determining was performed as previously described^30^. In order to investigate the interactions of THI and other seven pesticides, their combinations were conducted at an equitoxic ratio (50% of the 4-days LC_50_ of each pesticide). The total concentration of each blend was methodically changed, and all the above-mentioned proportions were constant to elucidate the concentration–response relationship. All experiments were carried out three times for each concentration.

### Statistical analysis

Determined results were analyzed with SAS probit (SAS Institute, Cary, NC). The LC_50_ data were calculated, and corresponding numerical values were deemed obviously different if their corresponding 95% confidence intervals (CIs) did not overlap.

To evaluate the mixture toxicity, the additive index data (AID) was computed according to the LC_50_ data of single pesticides and their combinations^31^. This method states an AID for the combined effect of a pesticide mixture. The biological activity (*S*) of a mixture consisting of pesticides *A* and *B* was tested by the equation as follows:

$$S = \, \left( {Am/Ai} \right) \, + \, \left( {Bm/Bi} \right),$$

where *A* and *B* express the different chemicals; *i* is the LC_50_ data for *A* or *B* individually; *m* is the LC_50_ data of *A* or *B* in the combination; and *S* expresses the sum of biological activity. Then *S* values were adopted to compute the AID adopting the following formula:

$${\text{AID }} = \, \left( {{1}/S} \right) - {\text{ 1 for}}\;S \le { 1}.0,{\text{ and AID }} = {1 } - S\;{\text{for}}\;S > { 1}.0.$$

Combined toxicities were ranked as antagonism (AID ≤ − 0.2), additive action (− 0.2 < AID ≤ 0.25) or synergism (AID > 0.25) accordingly^32^.

### Ethics approval and consent to participate

The authors confirm that the national laws regarding animal protection were followed.

## Results

### Toxic effects of individual chemicals

The toxicity to *A. mellifera* was highly variable among different classes of pesticides and among pesticides within the same class. Besides, each pesticide exhibited different toxicities with different treatment durations (Table 1). For 2-days treatment, the LC_50_ data of the detected chemicals to the insects from the range of 0.53 (0.47–0.62) to 1343 (1086–1780) μg mL^−1^. The rank of the toxicity for eight compounds was: THI > DIM, MET > PER > ZCY > TET, CYF > ESF. Among these pesticides, the feeding toxicity of THI was the highest with a LC_50_ data of only 0.53 (0.47–0.62) μg mL^−1^, followed by DIM and MET with LC_50_ data of 9.01 (7.79–10.77) and 12.50 (9.94–17.11) μg mL^−1^, respectively. Conversely, ESF showed the least toxicity with a LC_50_ of 1343 (1086–1780) μg mL^−1^. Therefore, THI, DIM and MET were 2534, 149 and 107 times higher than EST, respectively. For 4-days treatment, the LC_50_ data of evaluated chemicals to the insects from the range of 0.33 (0.28–0.38) to 1,212 (988.4–1573) μg mL^−1^. The rank of the toxicity for eight chemicals was: THI > DIM, MET > PER > ZCY > CYF, TET > ESF. Among the examined pesticides, THI still exerted the greatest toxicity with a LC_50_ data of 0.33 (0.28–0.38) μg mL^−1^, followed by DIM and MET with LC_50_ data of 6.54 (5.78–7.49) and 6.59 (5.51–8.07) μg mL^−1^, respectively. Contrarily, ESF still possessed the least toxicity with a LC_50_ data of 1,212 (988.4–1573) μg mL^−1^. Based on their LC_50_ data, THI, DIM and MET were 3,673, 185 and 184 times higher than ESF, respectively. For 7-days treatment, the LC_50_ data of the evaluated chemicals to the pollinators from the range of 0.25 (0.20–0.29) to 1125 (922.4–1442) μg mL^−1^. The rank of the toxicity for eight compounds was: THI > MET, DIM > PER > ZCY, CYF ≥ TET > ESF. Among the evaluated pesticides, THI still elicited the highest toxicity with a LC_50_ data of 0.25 (0.20–0.29) μg mL^−1^, followed by MET and DIM with LC_50_ data of 4.19 (3.58–4.88) and 5.30 (4.65–6.03) μg mL^−1^, respectively. Conversely, ESF still showed the least toxicity with a LC_50_ data of 1125 (922.4–1442) μg mL^−1^. Based on their LC_50_ data, THI, MET and DIM were 4500, 268 and 212 times higher than ESF, respectively.

**Table 1:** Acute toxicity of eight pesticides to honey bee workers at different duration, expressed as median lethal concentration (LC_50_: μg mL^−1^).

Pesticides	2 days interval		4 days interval		7 days interval	
	Slope (SE)	LC_50_ (95% CI) μg mL^−1^	Slope (SE)	LC_50_ (95% CI) μg mL^−1^	Slope (SE)	LC_50_ (95% CI) μg mL^−1^
THI	2.92 (0.25)	0.53 (0.47–0.62)	2.91 (0.24)	0.33 (0.28–0.38)	3.60 (0.35)	0.25 (0.20–0.29)
TET	3.30 (0.29)	312.7 (271.2–356.6)	3.64 (0.34)	258.7 (221.2–295.3)	3.77 (0.36)	216.8 (180.7–250.2)
ZCY	3.25 (0.28)	182.9 (157.5–209.1)	3.60 (0.33)	156.9 (133.4–179.6)	3.77 (0.35)	149.8 (126.6–171.6)
DIM	3.35 (0.31)	9.01 (7.79–10.77)	3.73 (0.34)	6.54 (5.78–7.49)	3.48 (0.31)	5.30 (4.65–6.03)
MET	2.34 (0.23)	12.50 (9.94–17.11)	2.03 (0.18)	6.59 (5.51–8.07)	2.52 (0.21)	4.19 (3.58–4.88)
CYF	2.65 (0.23)	321.6 (274.3–388.5)	2.93 (0.25)	245.6 (213.4–286.0)	2.89 (0.25)	182.2 (157.9–209.9)
PER	2.39 (0.21)	82.07 (68.81–101.7)	2.77 (0.23)	51.82 (44.86–60.31)	3.68 (0.34)	33.78 (29.12–38.39)
ESF	2.28 (0.22)	1343 (1086–1780)	2.18 (0.21)	1212 (988.4–1573)	2.12 (0.20)	1125 (922.4–1442)

*CI* confidence interval, *THI* Thiamethoxam, *TET* Tetraconazole, *ZCY Zeta*-cypermethrin, *DIM* Dimethoate, *MET* Methomyl, *CYF* Cyfluthrin, *PER* Permethrin, *ESF* Esfenvalerate.

The toxicities of THI, DIM, MET and PER to the insects for 4-days treatment were obviously higher than their corresponding toxicities for 2-days treatment. The toxicities of MET, CYF and PER to honey bees for 7-days treatment were obviously higher than their corresponding toxicities for 4-days treatment. Besides, the toxicities of all the assessed pesticides (except for ZCY and ESF) for 7-days treatment were obviously higher than their corresponding toxicities for 2-days treatment, implying that their toxicities were positively correlated with treatment duration. Among the determined chemicals, the toxicities of THI, MET and PER to the insects for 7-days treatment were 11.68, 2.98 and 2.43 times higher than their corresponding toxicities for 2-days treatment, respectively. Overall, THI elicited the highest toxicity, followed by DIM and MET, while the pyrethroids and TET elicited the relatively low toxicities to honey bees.

### Toxic effects of pesticide combinations

To explicit the joint toxic effect of THI and other seven pesticides toward honey bees, the LC_50_ data of different pesticide combinations for 2-, 4- and 7-days treatments were examined.

#### Joint toxic effects of binary and ternary combinations

**Figure 1:**
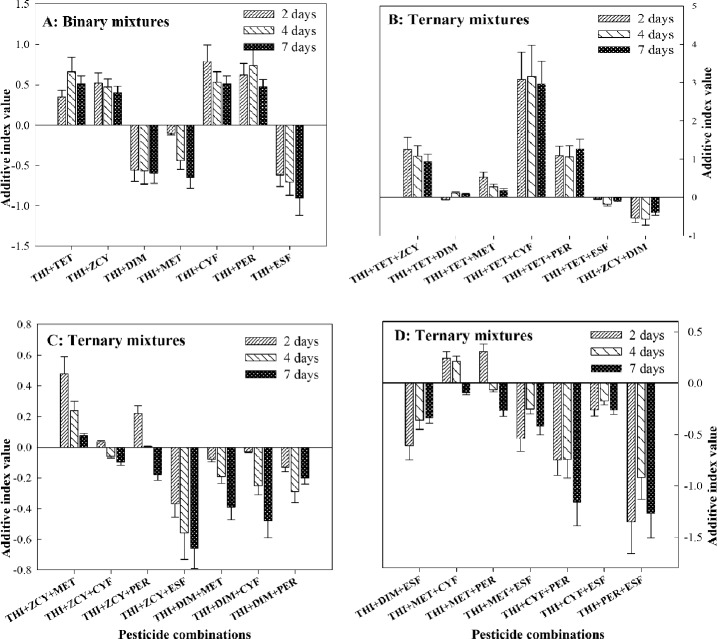
Joint toxic effects of binary and ternary combinations of thiamethoxam (THI) mixed with seven pesticides on honey bees. *CI* confidence interval, *THI* Thiamethoxam, *TET* Tetraconazole, *ZCY Zeta*-cypermethrin, *DIM* Dimethoate, *MET* Methomyl, *CYF* Cyfluthrin, *PER* Permethrin, *ESF* Esfenvalerate.

Four binary combinations of THI + TET, THI + ZCY, THI + CYF and THI + PER had synergistic actions on *A. mellifera*, and their AID from the range of 0.35 to 0.79 at 2-days treatment, from 0.47 to 0.74 at 4-days treatment, and from 0.40 to 0.51 at 7-days treatment. On the contrary, two binary combinations of THI + DIM and THI + ESF elicited antagonistic actions with their AID from the range of − 0.62 to − 0.56 at 2-days treatment, from − 0.71 to − 0.57 at 4-days treatment, and from − 0.90 to − 0.60 at 7-days treatment. The calculated AID of THI + MET at 2-d, 4-days and 7-days treatments were − 0.10, − 0.44 and − 0.65, respectively, implying additive and antagonistic actions on the pollinators (Fig. 1A).

Three ternary combinations of THI + TET + ZCY, THI + TET + CYF and THI + TET + PER elicited synergistic actions with AID from the range of 1.09 to 3.09 at 2-days treatment, from 1.06 to 3.17 at 4-days treatment, and from 0.95 to 2.97 at 7-days treatment. Contrarily, six ternary combinations of THI + ZCY + DIM, THI + ZCY + ESF, THI + DIM + ESF, THI + MET + ESF, THI + CYF + PER and THI + PER + ESF exhibited antagonistic actions on the insects with AID from the range of − 1.35 to − 0.37 at 2-days treatment, from − 0.92 to − 0.25 at 4-days treatment, and from − 1.427 to − 0.34 at 7-days treatment. Nonetheless, five ternary combinations of THI + TET + DIM, THI + TET + ESF, THI + ZCY + CYF THI + ZCY + PER and THI + MET + CYF showed additive actions, and their AID from the range of − 0.05 to 0.24 at 2-days treatment, from − 0.18 to 0.21 at 4-days treatment, and from − 0.18 to 0.092 at 7-days treatment. The other 11 ternary combinations exerted dual actions of joint toxic effects, such as synergistic-additive, synergistic-antagonistic and additive-antagonistic actions against honey bees (Fig. 1B–D).

#### Joint toxic effects of quaternary combinations

**Figure 2:**
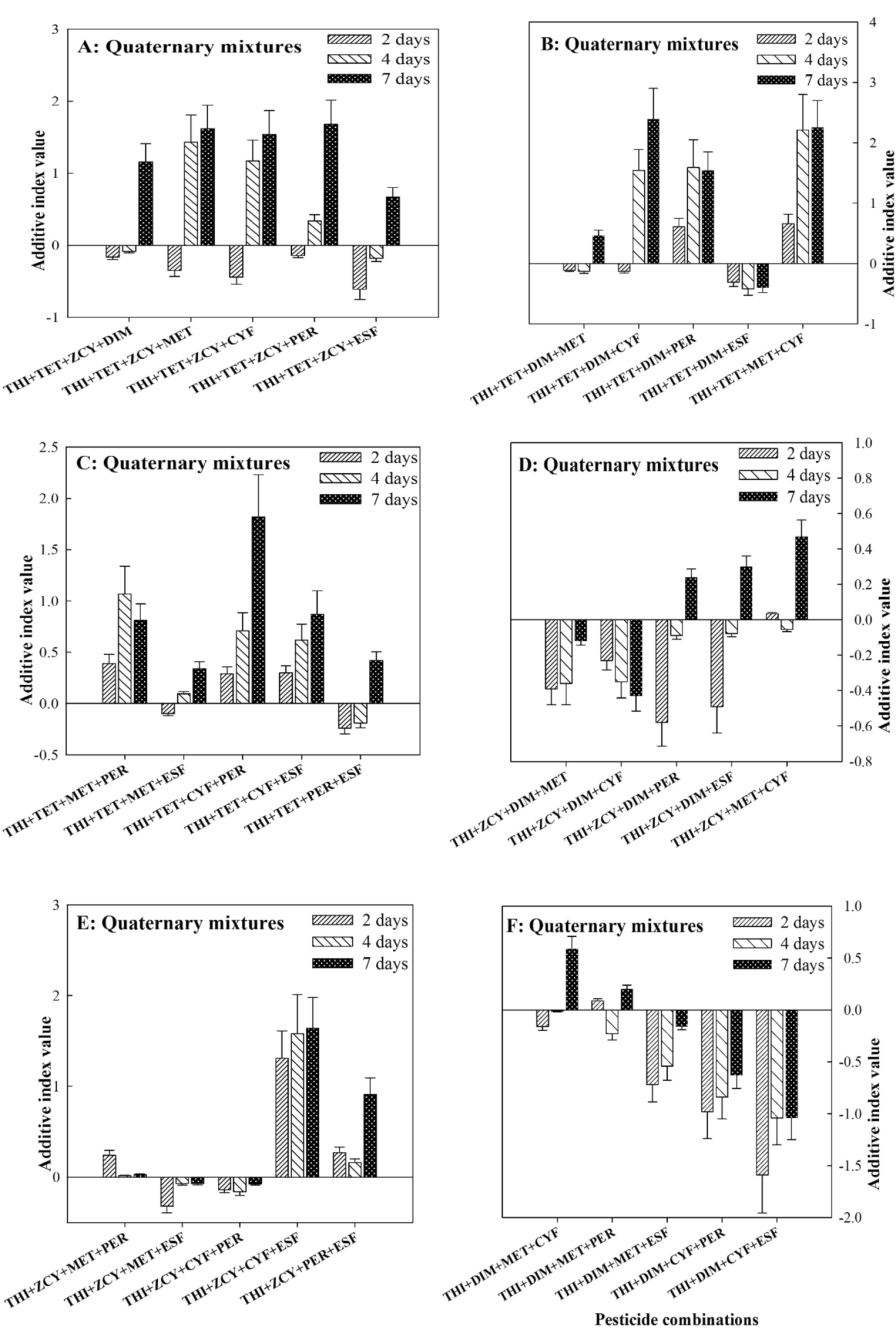

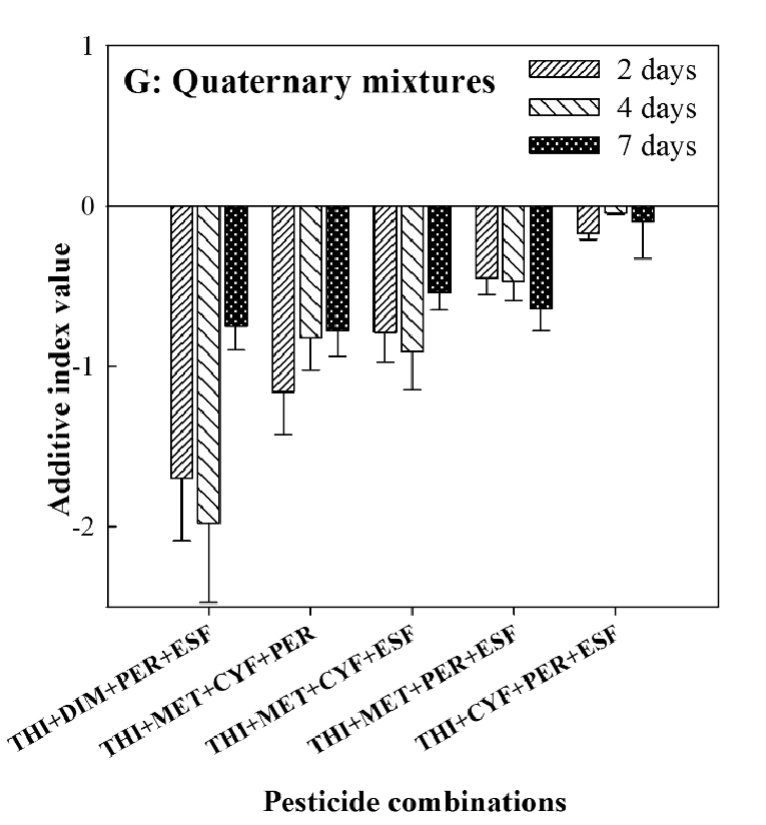
Joint toxic effects of quaternary combinations of thiamethoxam (THI) mixed with seven pesticides on honey bees. *CI* confidence interval, *THI* Thiamethoxam, *TET* Tetraconazole, *ZCY Zeta*-cypermethrin, *DIM* Dimethoate, *MET* Methomyl, *CYF* Cyfluthrin, *PER* Permethrin, *ESF* Esfenvalerate.

Six quaternary combinations of THI + ZCY + DIM + PER, THI + TET + MET + CYF, THI + TET + MET + PER, THI + TET + CYF + PER, THI + TET + CYF + ESF and THI + ZCY + CYF + ESF elicited synergistic actions on the insects with AID from the range of 0.29 to 1.31 at 2-days treatment, from 0.62 to 2.21 at 4-days treatment, and from 0.81 to 2.25 at 7-days treatment. Nonetheless, eight quaternary combinations of THI + TET + DIM + ESF, THI + ZCY + DIM + CYF, THI + DIM + CYF + PER, THI + DIM + CYF + ESF, THI + DIM + PER + ESF, THI + MET + CYF + PER, THI + MET + CYF + ESF and THI + MET + PER + ESF displayed antagonistic actions on the insects with AID from the range of − 1.70 to − 0.23 at 2-days treatment, from − 1.98 to − 0.35 at 4-days treatment, and from − 1.04 to − 0.40 at 7-days treatment. Nonetheless, the calculated AID of THI + ZCY + MET + PER, THI + ZCY + CYF + PER and THI + CYF + PER + ESF from the range of − 0.17 to 0.24 at 2-days treatment, from − 0.16 to 0.015 at 4-days treatment, and from − 0.096 to 0.027 at 7-days treatment, implying additive actions on *A. mellifera*. The other eight quaternary combinations elicited dual or triple actions of joint toxicity with different treatment durations (Fig. 2A–G).

#### Joint toxic effects of quinquenary to octonary combinations

**Figure 3:**
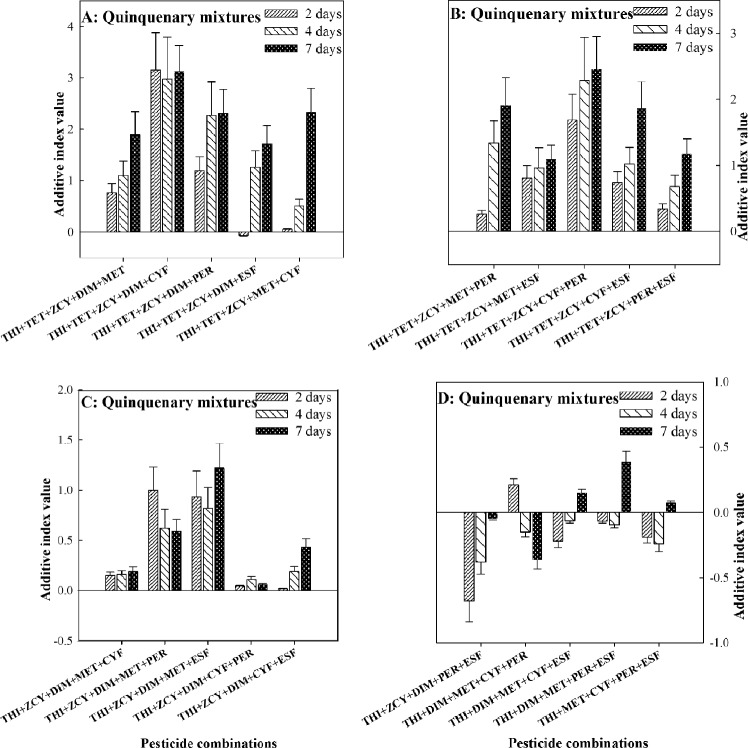
Joint toxic effects of quinquenary combinations of thiamethoxam (THI) mixed with seven pesticides on honey bees. *CI* confidence interval, *THI* Thiamethoxam, *TET* Tetraconazole, *ZCY Zeta*-cypermethrin, *DIM* Dimethoate, *MET* Methomyl, *CYF* Cyfluthrin, *PER* Permethrin, *ESF* Esfenvalerate.

Except for THI + TET + ZCY + DIM + ESF and THI + TET + ZCY + MET + CYF, all the quinquenary combinations incorporating THI and TET displayed synergistic actions on the bees, with their AID from the range of 0.26 to 3.15 at 2-days treatment, from 0.62 to 2.97 at 4-days treatment, and from 0.59 to 3.12 at 7-days treatment. The calculated AID of THI + ZCY + DIM + MET + CYF and THI + ZCY + DIM + CYF + PER from the range of 0.043 to 0.15 at 2-days treatment, from 0.11 to 0.16 at 4-days treatment, and from 0.058 to 0.19 at 7-days treatment. These findings suggested additive actions toward *A. mellifera*. Nonetheless, the other eight quinquenary combinations elicited dual actions of joint toxicity with different treatment durations (Fig. 3A–D).

**Figure 4:**
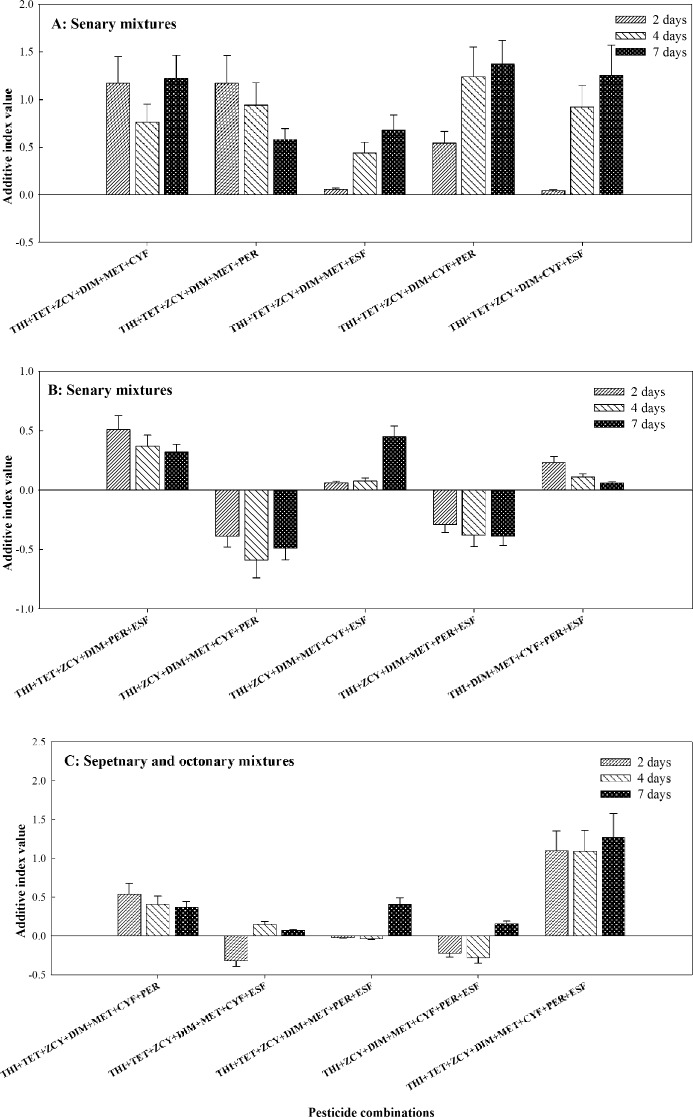
Joint toxic effects of senary combinations of thiamethoxam (THI) mixed with seven pesticides on honey bees. *CI* confidence interval, *THI* Thiamethoxam, *TET* Tetraconazole, *ZCY Zeta*-cypermethrin, *DIM* Dimethoate, *MET* Methomyl, *CYF* Cyfluthrin, *PER* Permethrin, *ESF* Esfenvalerate.

Except for THI + TET + ZCY + DIM + MET + ESF and THI + TET + ZCY + DIM + CYF + ESF, all the senary combinations incorporating THI and TET exhibited synergistic actions with AID from the range of 0.51 to 1.17 at 2-days treatment, from 0.37 to 1.24 at 4-days treatment, and from 0.32 to 1.37 at 7-days treatment. The calculated AID of THI + DIM + MET + CYF + PER + ESF at 2-days, 4-days and 7-days treatments were 0.23, 0.11 and 0.059, respectively, indicating additive actions against the pollinators. However, the other three senary combinations of THI + TET + ZCY + DIM + MET + ESF, THI + TET + ZCY + DIM + MET + ESF and THI + ZCY + DIM + MET + CYF + ESF had additive-synergistic actions on *A. mellifera* (Fig. 4A,B).

The septenary combination of THI + TET + ZCY + DIM + MET + CYF + PER exhibited AID of 0.54, 0.41 and 0.37 at 2-days, 4-days and 7-days treatments, respectively, implying synergistic actions on honey bees. On the contrary, two septenary combinations of THI + TET + ZCY + DIM + MET + CYF + ESF and THI + ZCY + DIM + MET + CYF + PER + ESF displayed additive-antagonistic actions with AID from the range of − 0.32 to − 0.22 at 2-days treatment, from − 0.28 to 0.15 at 4-days treatment, and from 0.072 to 0.16 at 7-days treatment. The calculated AID of THI + TET + ZCY + DIM + MET + PER + ESF at 2-days, 4-days and 7-days treatments were − 0.022, − 0.035 and 0.41, respectively, implying additive and synergistic actions toward *A. mellifera*. The octonary combination of THI + TET + ZCY + DIM + MET + CYF + PER + ESF exhibited AID of 1.10, 1.09 and 1.27 at 2-days, 4-days and 7-days treatments, respectively, implying synergistic actions on honey bees (Fig. 4C).

### Statistics of combination actions

#### Interaction patterns of binary and ternary combinations

**Figure 5:**
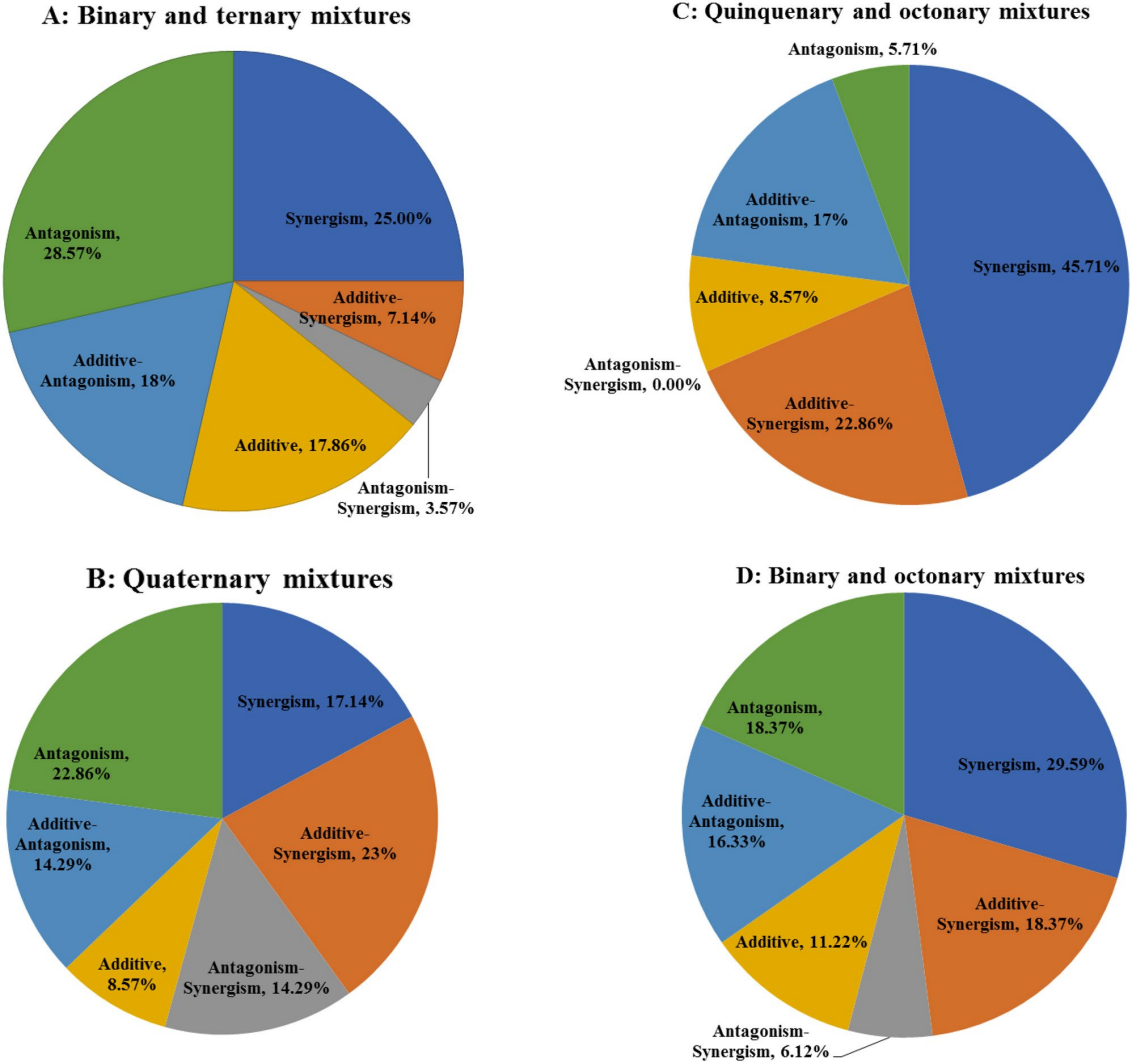
Interaction patterns of thiamethoxam (THI) mixed with seven pesticides on honey bees.

Add up to 28 THI-containing binary and ternary combinations were assessed in the present study. Briefly, 25% of binary and ternary combinations elicited synergistic actions on the insects. Additionally, 7.14% and 3.57% of combinations elicited synergistic-additive and synergistic-antagonistic actions, respectively. By comparison, 28.57% of pesticide combinations exerted antagonistic actions on *A. mellifera*. Both additive-antagonistic and additive actions were detected from 17.86% of combinations (Fig. 5A).

#### Interaction patterns of quaternary combinations

Add up to 35 THI-containing quaternary combinations were assessed in our study. Briefly, 17.14% of quaternary combinations presented synergistic actions. Additionally, 22.86% and 14.29% of quaternary combinations displayed additive-synergistic and antagonistic-synergistic actions on the insects, respectively. By comparison, 22.86% and 14.29% of combinations had antagonistic and additive-antagonistic actions, respectively. Only 8.57% of combinations exhibited additive actions toward the pollination insects (Fig. 5B).

#### Interaction patterns of quinquenary to octonary combinations

Add up to 35 THI-containing quinquenary to octonary combinations were evaluated in this study. Briefly, 45.71% of quinquenary to octonary combinations displayed synergistic actions. Besides, 22.86% of combinations elicited additive-synergistic actions on the insects. By comparison, 5.71% and 17% of combinations exhibited antagonistic and additive-antagonistic actions, respectively. Only 8.57% of combinations elicited additive actions against the insects (Fig. 5C).

Overall, 98 THI-containing binary to octonary combinations were examined in this assay. We exhibited that 29.59% of combinations displayed synergistic actions on the insects. Additionally, 18.37% and 6.12% of combinations elicited additive-synergistic and antagonistic-synergistic actions, respectively. By comparison, 18.37% and 16.33% of combinations exhibited antagonistic and additive-antagonistic actions on *A. mellifera*, respectively. Only 11.22% of combinations elicited additive actions. Additionally, 54.8% pesticide combinations incorporating THI and TET displayed synergistic actions against the insects. Above-mentioned findings suggested that 54.76% of pesticide combinations incorporating THI and TET elicited synergistic actions (Fig. 5D).

## Discussion

The success of neonicotinoids is mainly attributed to their systemic action^4^. Besides, the tested DIM, MET and TET also belong to systemic pesticides^33^. Therefore, these pesticides are often used for seed-coating and soil treatment globally^34^. Systemic chemicals can transfer from the soil, where they are used as granules or seed-coatings, through the sap of the plants and reach the nectar glands at the time of pollination, when the pollinators are attracted to the flowers^35,36^. Foraging bees are contacted mainly to systemic compounds via ingestion of contaminated nectar following seed coating and soil treatment^37^. Moreover, the application of systemic neonicotinoids differs from the classical spraying pesticides, which are remain on the plant only several hours or days after utilization^4^. With systemic neonicotinoids, the exposure of honey bees to THI possibly lasts several weeks during flowering^38^. Especially, repeated consumption of polluted nectar or pollen inside the hive can present either immediate or delayed effects^39^. Some reports have also shown that feeding exposure is often the most relevant and conservative approach for honey bees^40^. Therefore, we detected the joint toxic effects of THI and other pesticides on *A. mellifera* through feeding toxicity test.

Considering the greatly controlled circumstances and precise laboratorial design, the LC_50_ values from acute toxicity determinations become worthy if they can be adopted to forecast the influences of compounds on the pollinators under field environments^21,28^. The procured LC_50_ values of pesticides in our present research were almost unlikely in the ecosystem, and such values can only be happened in a specific case, such as direct usage or unlucky leakage^23,24^. Nonetheless, there is no assurance that the bee population could exposure to these pesticides inside this range of levels^10,37^. Results from assays of individual compounds elucidated that THI elicited the highest toxicity, followed by DIM and MET. In contrast, pyrethroids and TET exhibited the least toxicities, showing variable toxicity responses. Some studies have demonstrated that the oral toxicity (48-h LC_50_) and contact toxicity (topical application) (24-h LC_50_) of THI to *A. mellifera* are 0.13 μg mL^−1^ and 0.0299 μg bee^−1^, respectively, indicating that it is a highly toxic compound to *A. mellifera*^41,42^. Honey bees are greatly affected by THI because the compound can be rapidly converted to clothianidin, which has a high affinity for the insect nAChR and may contribute to bee mortality^16^. DIM and MET also have great toxicities to the bees with spray toxicity test^28^. Besides, the contact toxicity (topical application) (48-h LC_50_) of DIM is 0.1 μg mL^−1^, and it can be used as a toxic reference in test guideline of pesticide to honey bees^43,44^. The strong toxicities of DIM and MET may be contributed to dramatically reduced capacity to degrade them in honey bees^16^. Therefore, more concern should be paid to evaluate the utilization of THI, DIM and MET in integrated pest management (IPM) programs due to their detrimental severe influences on honey bees.

Pyrethroids are not systemic pesticides and do not have translaminar actions, so they are usually formulated with systemic neonicotinoids as mixtures in order to broaden the insecticidal spectrum and delay resistance by pests^45^. Three binary mixtures of THI + ZCY, THI + CYF and THI + PER exhibited the synergistic effects on *A. mellifera*. Synergistic interaction can hazard non-target organisms, which is undesirable in natural ecosystems^22^. If one chemical in the mixture causes an alteration in toxicokinetics in the organism, synergistic effects can be found in many mixtures^27^. One possible interpretation for the synergy in the co-occurrence of THI and different pyrethroids could be related to the metabolism competition mediated by P450 catalytic sites^16^. However, we found that the combination of THI + ESF exerted antagonistic effects on honey bees. Therefore, it is urgently necessary to assess the potential interaction between pyrethroids and neonicotinoids as well as the underlying detoxification mechanism, which would help us evaluate whether novel or existing compounds could be considered safe for honey bees^46^.

The agricultural use of fungicides has been dramatically increased over the past decade to control fungal outbreaks^24^. Fungicides are the most abundant plant protection products monitored in bees and bee products, since these compounds can be used during bloom when honey bees are foraging^16^. Although fungicides usually seem safe to honey bees, these compounds may, in certain circumstance, cause adverse effects^22^. Neonicotinoids are frequently co-applied with various fungicides during farming practice to afford a broader spectrum of management with fewer utilization relative to individual compounds^46^. Our findings revealed that most of the pesticide combinations incorporating THI and TET exerted synergistic effects on the insects, and therefore, it might pose a greater-than-expected hazard to pollination insects^26^. In other words, the synergistic interaction between THI and TET could transcend the effect of the additive or antagonistic interactions among other pesticide combinations. This finding has also been disclosed in other studies, in which the synergistic interactions between diazinon and fenitrothion or thiobencarb are able to mask the influence of the antagonistic interaction between fenitrothion and thiobencarb^47^. The biochemical mechanism of this synergism might be attributed to an interaction between TET and the cytochrome P-450 monooxygenase system, which is responsible for detoxifying THI^16,41^. Knowledge of the enzymes and inhibitors in neonicotinoid metabolism would facilitate the safe and effective application of these pesticides^3^. Consequently, the effects of the combined exposure to mixtures containing THI and TET should be considered in risk assessment determinations for alleviating the side-effects on *A. mellifera*.

Many reports have demonstrated that honey bees living in agricultural landscapes are conventionally contacted to pesticide conbinations^15,26^. However, it remains largely unknown about the mixture toxicity to bees in these situations^29,41^. The widespread detection of agrochemical mixtures in bee tissues enhances attentions about the potential harmful influences of concurrent exposure to a cocktail of chemicals^24^. Generally, only the impacts of individual toxicants are assessed both for research and pesticide registration protocols, and exposure to combinations are only determined in risk evaluations when they are part of the same formulation^33^. However, the application of two or more pesticides is a common practice in conventional farming during the same cropping season, and hence complex pesticide combinations which are not co-formulants of an indivdual product can be concurrently discovered in bee forage^15,25^. This issue is worrisome given that exposure to combinations might have higher risks to bee health than the individual influence of a specific class of chemicals^22,26,46^. In recent years, the majority of concern to neonicotinoid toxicity has been concentrated on their persistence in the environment and potential toxicity risks to bees^4,6^. Apart from lethal effects, some reports have demonstrated that exposure to field-realistic concentrations of neonicotinoids can exert sub-lethal effects on the bees^7^. Therefore, it is necessary to conduct chronic determinations for pesticide exposure in the pollination insects^8^. Besides, some studies have shown that neonicotinoids have time-dependent and time-cumulative effects, so that the risk of foraging bees feeding on small levels of residues becomes an unignorable issue^48^. This means that these pesticides can cause effects at any level if the exposure duration is sufficient^49^. Therefore, the traditional risk assessment method can not predict the influences of neonicotinoids on the environment^8^. A new risk evaluation system is needed to determine the effects of such time-dependent pesticides on crop pollinators and ecosystems.

## Conclusions

Among the eight tested pesticides, THI elicited the highest toxicity, followed by MET and DIM, whereas pyrethroids and TET elicited relatively low toxicities to honey bees. Among 98 examined THI-containing binary to octonary mixtures, 29.59% of combinations exhibited synergistic effects. Because many types of pesticides may co-occur in natural ecosystem, it is important to detect pesticide interactions with crop pollinators. Determination of only single chemicals could underrate the actual environmental risk. Hence, synergistic effects of pesticide mixtures must be cautiously taken into account to mitigate side-effects on pollination insects and keep effective control toward harmful organisms.

## Data Availability

The datasets used or analysed during the current study are available from the corresponding author on reasonable request.
